# Genome Instability and Long Noncoding RNA Reveal Biomarkers for Immunotherapy and Prognosis and Novel Competing Endogenous RNA Mechanism in Colon Adenocarcinoma

**DOI:** 10.3389/fcell.2021.740455

**Published:** 2021-10-20

**Authors:** Ziyuan Ren, Zhonglin Wang, Donghong Gu, Hanchen Ma, Yan Zhu, Menghua Cai, Jianmin Zhang

**Affiliations:** ^1^Department of Immunology, CAMS Key Laboratory for T Cell and Cancer Immunotherapy, Institute of Basic Medical Sciences, Chinese Academy of Medical Sciences and School of Basic Medicine, Peking Union Medical College, State Key Laboratory of Medical Molecular Biology, Beijing, China; ^2^Cheeloo College of Medicine, Shandong University, Jinan, China; ^3^School of Physical Science, University of California, Irvine, Irvine, CA, United States; ^4^Weihai Municipal Hospital, Cheeloo College of Medicine, Shandong University, Weihai, China; ^5^Department of Translational Molecular Pathology, The University of Texas MD Anderson Cancer Center, Houston, TX, United States

**Keywords:** genome instability, lncRNA, ceRNA, immune checkpoint inhibitor, MSI, tumor heterogeneity, colon adenocarcinoma

## Abstract

**Background:** Long noncoding RNAs (lncRNAs) crucially modulate DNA damage responses/repair in cancer cells. However, the underlying regulatory role of genome integrity and its clinical value in colon adenocarcinoma (COAD) remains unclear. This study links genome instability to lncRNA using computational biology techniques, in attempt to propose novel biomarkers of immunotherapy outcome, and investigated a potential competing endogenous RNA (ceRNA) as a molecular regulatory mechanism.

**Methods:** TCGA-COAD patients were divided into genome unstable (GU)-like and genome stable (GS)-like clusters *via* hierarchical clustering to predict immunotherapy outcomes. Multivariate Cox model was established to predict the overall survival rate in COAD patients. Additionally, SVM and LASSO algorithms were applied to obtain hub lncRNAs. A novel genome instability-related ceRNA network was predicted with the Starbase 2.0 database. To better understand how these genes fundamentally interact during tumor progression and development, the mutation analysis and single-gene analysis for each gene was performed.

**Results:** In contrast to those in the GS-like cluster, GU-like-cluster patients demonstrated a higher tumor mutational burden (TMB)/microsatellite instability (MSI), DNA polymerase epsilon (*POLE*) mutation rate, and immune checkpoint expression, all indicate a greater predictive power for response rate for immunotherapy. The novel prognostic signature demonstrated an outstanding predictive performance (AUC > 0.70). The genes in the genome insatiability-related ceRNA network (including four axes: *AL161772.1-has-miR-671-5p* (*hsa-miR-181d-5p*, *has-miR-106a-5p*)-*NINL*, *AL161772.1-has-miR-106a-5p-TNFSF11*, *AC124067.4-hsa-miR-92b-3p* (*hsa-miR-589-5p*)-*PHYHIPL*, and *BOLA3-AS1-has-miR-130b-3p-SALL4*) were identified as critical regulators of tumor microenvironment infiltration, cancer stemness, and drug resistance. qPCR was performed to validate the expression patterns of these genes. Furthermore, the MSI-high proportion was greater in patients with mutated type than in those with the wild type according to all four target genes, indicating that these four genes modulate genomic integrity and could serve as novel immunotherapy biomarkers.

**Conclusion:** We demonstrated that genome instability-related lncRNA is a novel biomarker for immunotherapy outcomes and prognosis. A novel ceRNA network that modulates genomic integrity, including four lncRNA-miRNA-mRNA axes, was proposed.

## Introduction

Colon adenocarcinoma (COAD) is the primary subtype of colorectal cancer, ranking fourth in lethal malignancies worldwide and second across the United States (Brody, 2015). Although the establishment of a series of molecularly targeted therapies has led to a noticeable increase in the survival of COAD patients, chemotherapy is still the standard and irreplaceable treatment, and the 5-year survival rate for Stage IV COAD patients is less than 10% (Ganesh et al., 2019). Thus, there is an urgency to further develop novel treatment regimens (Garrido-Castro et al., 2019).

In recent years, immunotherapy has become an ideal option for advanced COAD with the emergence and rapid development of immune checkpoint inhibitors (ICIs) (Qin et al., 2019; Hu et al., 2020). Considering that patient response rates to nivolumab and pembrolizumab (both PD-1/PD-L1 inhibitors) are diverse and often less than 50%, additional predictive biomarkers need to be identified (Schrock et al., 2019). Genome instability was reported as one of the top 10 promising discoveries for cancer treatment in the twenty-first century and has elicited a corresponding interest (Hanahan and Weinberg, 2011). Recent clinical practice has demonstrated that genome instability is associated with ICI outcomes. Microsatellite instability (MSI) is a critical biomarker for ICIs (Boland and Goel, 2010). In general, *MLH1* promoter hypermethylation or germline mutations in four DNA mismatch repair (dMMR) machinery genes (*MSH6*, *MSH2*, *PMS2*, *MLH1*) lead to MSI (Dudley et al., 2016). PD-1/PD-L1 inhibitors in MSI-high (MSI-H) metastatic colorectal carcinoma have been confirmed to have a favorable cancer-control effect with high progression-free survival (Schrock et al., 2019).

Long noncoding RNAs (lncRNAs) are nonprotein coding RNAs that are longer than 200 nucleotides. They are involved in a series of diverse biological processes, including cell development, differentiation (Fatica and Bozzoni, 2014), the cell cycle response (Hung et al., 2011), and gene imprinting (Kanduri, 2016). Current studies on these molecules have mainly focused on their dysregulation in cancers, which leads to alterations in tumor behavior (Bhan et al., 2017). Therefore, lncRNAs are promising candidates for clinical cancer biomarker exploration (Li Y. et al., 2020; Goyal et al., 2021). A novel role for lncRNAs is modulating DNA damage response pathways, such as the TP53 and ATM/ATR pathways (Su et al., 2018). This study linked genome instability to lncRNA, which was termed genome instability-related lncRNA (GIRlncR), and hypothesized that GIRlncR could serve as a novel immunotherapy biomarker for COAD.

Advances in RNA sequencing (RNA-seq) techniques have largely promoted the functional annotation and progress on the computational characteristics of lncRNAs (Chen et al., 2016). Yin et al. (2021) identified a genome instability-related lncRNA prognostic signature in COAD using computational biological techniques. However, they did not focus enough on the predictive ability of GIRlncR for immunotherapy outcomes. They also did not explore the regulatory mechanisms of lncRNAs (Yin et al., 2021). This study established a patient stratification clustering method for COAD to predict ICI outcomes in these patients. A novel genome instability-related competing endogenous RNA (ceRNA) network (including four axes: *AL161772.1-has-miR-671-5p* (*hsa-miR-181d-5p*, *has-miR-106a-5p*)-*NINL*, *AL161772.1-has-miR-106a-5p-TNFSF11*, *AC124067.4-hsa-miR-92b-3p* (*hsa-miR-589-5p*)-*PHYHIPL*, and *BOLA3-AS1-has-miR-130b-3p-SALL4*) was constructed using machine learning algorithms. Furthermore, the MSI-H proportion was greater in patients with mutated type than in those with the wild type according to all four target genes, indicating that these genes modulate genomic integrity and could serve as novel immunotherapy biomarkers.

## Materials and Methods

### Data Selection and Code Availability

The graphical abstract presents the flow chart and online resources for this study. The original data of RNA-seq (FPKM format), miRNA-seq, corresponding clinical characteristics, including gender, age, overall survival (OS), and stage, and simple nucleotide variation (SNV) were downloaded from the TCGA-COAD project (December 24, 2020). The tumor mutational burden (TMB) and MSI data were retrieved from cBioportal (Cerami et al., 2012). The clustering method was used to detect and exclude the outlier RNA-seq samples. Eventually, 453 RNA-seq samples were acquired; however, only 446 patients with both matching RNA-seq data and corresponding clinical features were included in this study. All biological and clinical samples in this study are publicly available. The data availability policies of the open-accessed databases were strictly followed. All code utilized in this study can be acquired *via* email 201800413040@mail.sdu.edu.cn with reasonable grounds.

### Differential Expression Gene Extraction

The R package “limma” was used to extract differential expression genes (DEGs). All tumor RNA-seq samples were sorted according to their SNV numbers, from largest to smallest. The first 25% and last 25% of samples were named the genome-unstable (GU) group and genome-stable (GS) group. Differentially expressed lncRNAs between the GS and GU groups were considered GIRlncRs. The cutoff value was adjusted a *p*-Value (adj. *p*) < 0.05, and | log_2_ fold-change| > 1. Similarly, genome instability-related mRNA (GIRmR) and miRNA (GIRmiR) were extracted.

### Hierarchical Clustering to Establish a Novel Immunotherapy Outcome Predictive Stratification Method

The “sparcl” package was implemented to perform unsupervised hierarchical clustering based on GIRlncRs (*k* = 2). The clusters with higher and lower SNV numbers were named GU-like and GS-like clusters, respectively. We compared the tumor mutational burden (TMB), MSI, expression, and mutation rates of four MMR genes, expression of six immune-related genes, and the mutation rates of *POLE* (a novel biomarker for immunotherapy outcomes (Wang et al., 2019)) between the two clusters. The MMR and immune-related genes included in this study are listed in Table 1. Single-sample gene set enrichment analysis (ssGSEA) was used to compare tumor microenvironment (TME) infiltration and functions between the two clusters (Supplementary Table 1). Survival analysis using the log-rank test between the GS-like and GU-like clusters was performed to evaluate the prognostic value of clustering. By calculating the Pearson’s correlation coefficients, we ranked the relevance of associations between each mRNA and GIRlncR expression. The first 10 ranked mRNAs were considered coexpressed with GIRlncR. Cytoscape, a biological network modifying software, was used to visualize the lncRNA-mRNA coexpression network (Shannon et al., 2003). To explore the potential functions of GIRlncRs, functional gene enrichment analysis based on the Gene Ontology (GO) (Ashburner et al., 2000) and Kyoto Encyclopedia of Genes and Genomes (KEGG) (Kanehisa and Goto, 2000) databases was performed.

**TABLE 1:** The MMR genes and immune-related genes utilized in this study.

MMR genes	Immune-related genes
MSH2	PDCD1 (PD-1)
MSH6	CD274 (PD-L1)
PMS2	PDCD1LG2 (PD-L2)
MLH1	CTLA4
	CD80
	CD86

*MMR, mismatch repair.*

### Multivariate Cox Regression to Construct a Novel Gene Signature

Multivariate Cox regression is the most widely utilized regression model for analyzing medical survival time and survival status data (Bradburn et al., 2003), and it was used to explore the association with event incidence (surviving/deceased in this study). The hazard ratio (HR) represents the probability of event occurrence under the currently observed feature patterns (gene expression pattern in this study). First, we divided 446 patients equally into the training and test cohorts. Chi-square tests were used to detect selection bias for each feature during patient division. Second, univariate Cox regression analysis of each GIRlncR was used for feature selection. Only statistically significant features (*p* < 0.05) were used for model construction. Third, multivariate Cox regression was performed to construct the genome instability-related lncRNA prognostic signature (GIlncPS) using the training cohort. The constructed model is as follows:


Risk_score=Σ(geneexpression×coefficient)

HR=exp(coefficient)

The model was applied to the test cohort. In both training and test cohorts, patients who had a risk score higher than the median value of the risk score were classified as the high-risk group and vice versa. Survival analysis and receiver operating characteristic (ROC) curves and the area under the curve (AUC) were utilized to evaluate the reliability of the GIlncPS. Moreover, given that *BRAF* is a frequently mutated gene with prognostic value (Sanz-Garcia et al., 2017), we tested whether our signature has better predictive performance than this event. Survival analysis among *BRAF* mutation-type/high-risk, *BRAF* mutation-type/low-risk, *BRAF* wild-type/high-risk, AND *BRAF* wild-type/low-risk groups was performed using the log-rank test. To demonstrate that our signature has a better predictive performance than other lncRNA signatures, we found two proposed lncRNA signatures for COAD, namely, Li’s signature (Li Z. et al., 2020) and Jin’s signature (Jin et al., 2020). AUC was used to rank the predictive performance of these signatures according to OS at 1, 3, and 5 years for 446 patients. To demonstrate that our signature has prognostic ability independent of general factors, we performed multivariate Cox regression, including clinical features (age, gender stage, T (tumor), M (metastasis), N (lymph node), *KRAS* mutation type, *TP53* mutation type, *BRAF* mutation type, and *POLE* mutation type) and the risk score. The nomogram, including our signature, was plotted for clinical reference, which was evaluated using a calibration curve.

### Analysis of Mutation Profile and Identification of Hub-Long Noncoding RNAs in Genome Stable- and Genome Unstable-Like Clusters

To investigate the relationship between the clusters and risk groups, Sankey plots were constructed. Waterfall plots were generated to explore the diversity of mutation profiles between GS- and GU-like clusters. To extract lncRNAs that had the closest relationship with clustering, LASSO and SVM algorithms were applied. The lncRNAs included in LASSO-screened lncRNAs, SVM-screened lncRNAs, and prognostic signatures were considered hub lncRNAs.

### Competing Endogenous RNA Network

The hub-lncRNA and target mRNA interactions were predicted using an online comprehensive RNA database, ENCORI^1^ (Li et al., 2014). The overlapping mRNAs in GIRmRs and hub-lncRNA target mRNAs require further research. Only the statistically significant lncRNA-mRNA pairs (*p* < 0.05) in COAD remained for further research. We then retrieved the miRNAs mediating the relationships between the lncRNAs and mRNAs. The retrieved miRNAs that were differentially expressed between the GS and GU groups were retained for ceRNA network construction. Figure 4G illustrated the screening process of ceRNA network.

### Comprehensive Single-Gene Analysis

It is hypothesized that the genes included in the ceRNA network play a pivotal role in COAD. A comprehensive single-gene analysis was performed. Differential expression analysis was performed between normal and tumor tissues from COAD patients by employing the Mann-Whitney *U* test. Thorsson et al. (2018) identified six immune subtypes across 33 TCGA cancer types (C1–C6) and compared the expression of each gene among these six subtypes using the Kruskal-Wallis test. Spearman correlation analysis was performed to detect the correlation between each gene and TME infiltration, the expression of MMR genes, immune-related genes (Table 1), and two previously discovered crucial lncRNAs regulating genome instability (*NOARD* (Munschauer et al., 2018), *GUARDIN* (Hu et al., 2018)). The TME was evaluated by the estimation of stromal and immune cells in malignant tumor tissues using expression data (ESTIMATE) immune data, stromal score (Yoshihara et al., 2013), and cell-type identification by estimating relative subsets of RNA transcripts (CIBERSORT) and specific immune cell types (Newman et al., 2019). Given that genome instability contributes to tumor heterogeneity, which leads to drug resistance (Morel et al., 2017; Dagogo-Jack and Shaw, 2018; Sansregret et al., 2018), we performed cancer stemness and drug sensitivity analysis for these genes. Malta et al. (2018) identified a novel index to evaluate cancer stemness features based on DNA methylation patterns (DNA stemness score (DNAss)) and mRNA expression patterns (RNA stemness score (RNAss)). We calculated the Spearman correlation between each gene and cancer stemness. Drug sensitivity analysis was also performed using an open-access database, specifically National Cancer Institute (NCI)-60. The Kolmogorov-Smirnov test was applied to verify the normality of the included indexes, including TMB/MSI scores, NCI-60 indexes, TME indexes, and cancer stemness indexes (Supplementary Table 2). We found that the drug sensitivity index was normally distributed. Therefore, the method of Pearson’s correlation to explore the association between gene expression and drug sensitivity is appropriate.

### Expression Pattern Validation by qPCR

Total RNA was isolated from COAD cell lines named Caco-2, Lovo, HCT116, and HT29 using the RNeasy Mini Kit (QIAGEN, Hilden, Germany), according to the manufacturer’s instructions. Complementary DNA (cDNA) was produced by RNA using the PrimeScript^TM^ Reverse Transcription Kit (TakaRa, Maebashi, Japan) in an ABI 7500 System (Applied Biosystems, Thermo Fisher Scientific, Waltham, MA, United States). The primers specific for target genes were designed and synthesized by Tianyi Huiyuan Biotech (Beijing, China). The following procedures were performed: activation of enzymes at 50°C for 2 min and then 95°C for 2 min, 45 cycles of denaturation at 95°C for 15 s, annealing at 58°C for 20 s, and extension at 72°C for 30 s. The relative expression levels of the target genes were calculated using the 2^−ΔΔ*CT*^ method. Glyceraldehyde 3-phosphate dehydrogenase (GAPDH) was used as the internal control. All qPCR reactions were performed in triplicate. The primers used in this study are listed below.

**Table:** 

Genes	Primer sequence (5′–3′)
AC124067.4 (lncRNA)	Forward: ATGAGAGGGTTGGGTGCAAG Reverse: GCCTTTTCCTTGTGGCTGTG
AL161772.1 (lncRNA)	Forward: ATGCCCATGAACAGCCATGA Reverse: GGCTGTTGCTCCTTTCTCCT
BOLA3-AS1 (lncRNA)	Forward: AGTCAGAAGCTCCGAGGCTA Reverse: TTTGCGGACAGTTCTACCCC
SALL4	Forward: TCGATGGCCAACTTCCTTC Reverse: GAGCGGACTCACACTGGAGA
GAPDH	Forward: TGTTCGTCATGGGTGTGAAC Reverse: ATGGCATGGACTGTGGTCAT

### Mutation Analysis

Specific somatic mutations drive cancer development (Koch, 2017). Therefore, the mutation analysis of the target mRNAs was applied, including somatic mutations and copy number variation (CNV) analysis. The R package “RCircos” was used to explore the chromosome location and CNV number for each gene. We then explored the alteration rate, type, and site in the COAD datasets and examined the association between the alteration status and gene expression. We calculated the Spearman’s correlation TMB and MSI scores and assessed the expression of each gene to explore whether the target mRNAs were directly associated with genome instability. Furthermore, the association between target mRNA alteration status and MSI status was explored. All mutation analyses were performed using the online database cBioportal.

### Statistics

SPSS Statistics (version 26.0; IBM, Armonk, NY, United States) and Microsoft Excel (Microsoft Corporation, Redmond, WA, United States) were used to present the retrieved data and perform the Chi-square test. R language (version 4.0.3) was used to perform machine learning algorithms and correlation analysis (R Core Team, 2013). The Benjamini and Hochberg method was used to adjust the *p*-Value for DEG extraction.

## Results

### Differential Expression Genes Extraction and Hierarchical Clustering

**FIGURE 1:**
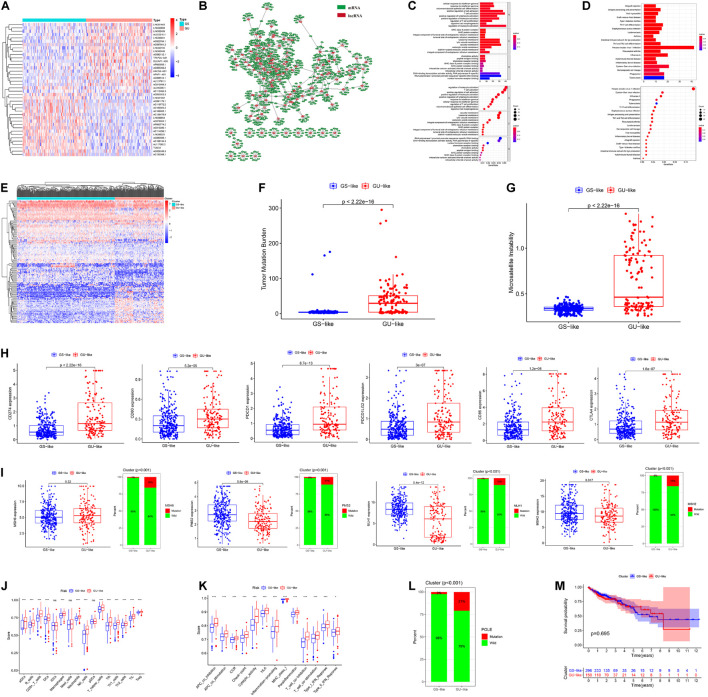
Hierarchical clustering and analysis of the genome stable (GS)-like and genome unstable (GU)-like clusters. **(A)** The heatmap of 123 genome instability-related lncRNAs (GIRlncRs) between the GS and GU groups. **(B)** The coexpression network of the GIRlncRs and top-10 correlated mRNAs for each lncRNAs by Pearson’s correlation. The functional enrichment analysis of the coexpressed mRNAs of GIRlncRs based on GO **(C)** and KEGG **(D)**. **(E)** The unsupervised hierarchical clustering based on 123 genome instability-related lncRNAs. The comparison of the tumor mutational burden **(F)**, microsatellite instability **(G)**, expression of six immune checkpoint-related genes (*CD274* (*PD-L1*), *CD80*, *PDCD1* (*PD-1*), *PDCD1LG2*(*PD-L2*), *CD86*, and *CTLA4*) **(H)**, expression and mutation rate of four DNA mismatch repair protein gene (*MLH1*, *MSH2*, *MSH6*, and *PMS2*) **(I)** and mutation rate of *POLE* **(L)** between the two clusters by Mann-Whitney *U* test. The comparison of the immune cell infiltration **(J)** and Immune functions **(K)** of the two clusters by ssGSEA. **(M)** The survival analysis of the two clusters by log-rank test. *p < 0.05; ***p < 0.001; and *ns*, not significant.

In total, 123 GIRlncRs were extracted between the GS and GU groups (Figure 1A). We constructed a mRNA and GIRlncR coexpression network (Figure 1B) to show the top 10 close interactions between each GIRlncR and corresponding mRNAs, indicating that the GIRlncRs regulate genome instability and complex tumor cell biological processes. Functional analysis further illustrated the relationship between GIRlncRs and tumor immunity (Figures 1C,D). Almost all GO- and KEGG-enriched pathways involved immune responses, such as antigen processing and T-cell activation. The unsupervised hierarchical clustering based on GIRlncRs was employed to divide COAD patients into two clusters with various genome instability statuses and immune therapy response rates. In total, 453 samples were clustered into GS-like (302 samples) and GU-like groups (151 samples; Figure 1E). Surprisingly, the TMB, MSI score, and expression of all immune checkpoint-related genes were higher in the GU-like cluster (*p* < 0.05), whereas expression of the three MMR genes (*MLH1*, *PMS2*, *MSH2*) was lower (*p* < 0.05; Figures 1F–I). The mutation rates of all four MMR genes were higher in the GU-like group (*p* < 0.05). The ssGSEA showed that almost all immune cells and immune functions were increased in the GU-like group (Figures 1J,K). Furthermore, the mutation rate of the novel immunotherapy response rate biomarker *POLE* was also significantly higher in the GU-like group (*p* < 0.05; Figure 1L). These findings strongly suggest that our clustering based on GIRlncRs could predict TME infiltration and ICI response rates in COAD patients. However, as shown in Figure 1M, the OS of the two clusters did not differ, indicating that the clustering method could not fulfill the purpose of prognosis.

### Multivariate Cox Regression to Construct the Genome Instability-Related LncRNA Prognostic Signature

**FIGURE 2:**
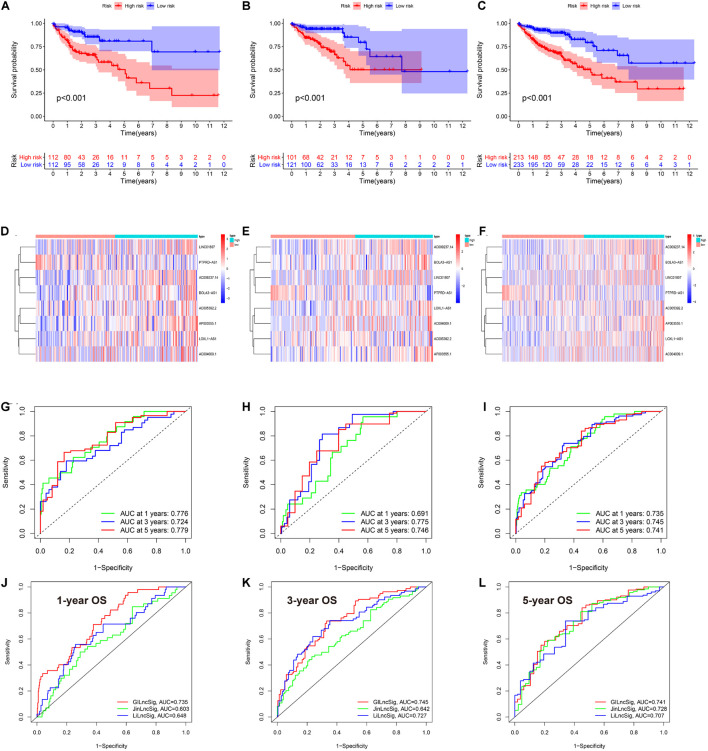
The eigth lncRNA prognostic signature in TCGA cohort. **(A–C)** Survival analysis between the high- and low-risk groups based on overall survival **(D–F)**. Heatmap of the eight lncRNA between high- and low-risk groups **(G–I)**. rROCeceiver operating characteristics curve of the signature model based on OS at 1-3-5-year. AUC, the area under the curve. **(A,D,G)** Training cohort; **(B,E,H)** are test cohort; **(C,F,I)** are TCGA cohort. Model comparison with two previously published lncRNA prognostic signatures by ROC curve based on overall survival in TCGA cohort at 1-**(J)**, 3-**(K)**, 5-**(L)** year.

**FIGURE 3:**
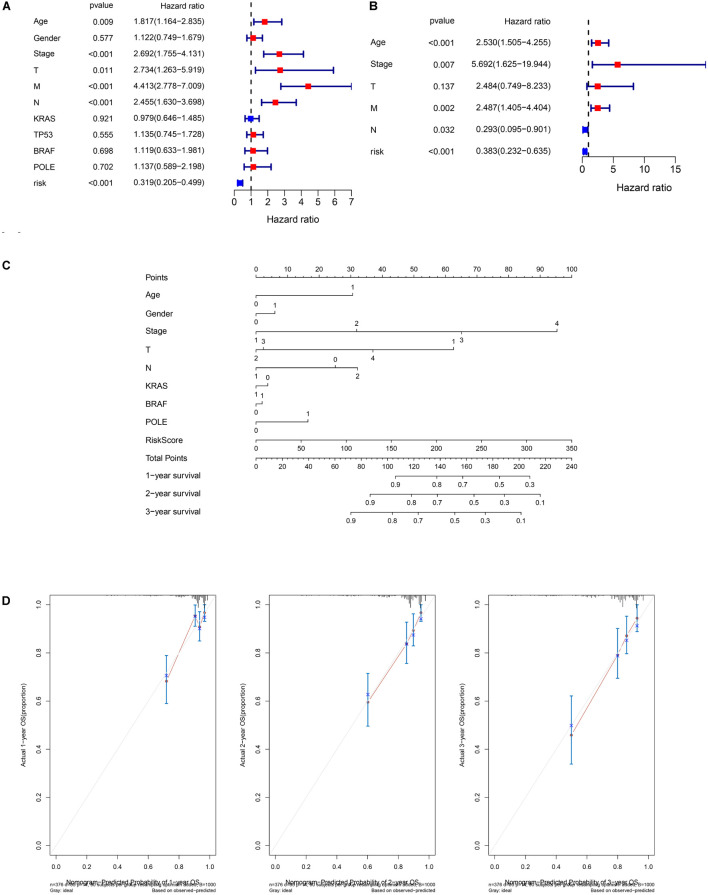
Independent prognostic analysis and nomogram of the 8-lncRNA prognostic signature. **(A)** Univariate Cox regression of the TCGA cohort. **(B)** Multivariate Cox regression of the TCGA cohort based on overall survival. Age (>65 vs. ≤65), gender (male vs. female), stage (III/IV vs. I/II), T (III/IV vs. I/II), M (I vs. 0), N (I/II vs. 0), *KRAS* (mutation vs. wild), *TP53* (mutation vs. wild), *BRAF* (mutation vs. wild), *POLE* (mutation vs. wild), and risk (low-risk vs. high-risk). **(C)** Nomogram of the TCGA cohort based on overall survival (M was not included in the nomogram due to only 20 patients with M1). **(D)** Calibration curve of the TCGA cohort based on overall survival at 1, 2, and 3 years.

Since our clustering could not predict OS in COAD patients, we constructed a novel GIlncPS. The basic characteristics of the training and test cohorts are shown in Supplementary Table 3. The Chi-square test showed that the division of training and test cohorts was proper without selection bias (all *p* > 0.05). The regression results are shown in Supplementary Table 4. An eight-lncRNA GIlncPS was acquired as follows: risk score = 0.239 × *LINC01807* + 0.235 × *AC009237.14* + 0.310 × *LOXL1-AS1* + 0.028 × *AC005392.2* + 0.751 × *AP003555.1* + 0.384 × *BOLA3-AS1* – 0.330 × *PTPRD-AS1* + 0.258 × *AC004009.1*. The high- and low-risk groups were divided according to median risk score. Survival analysis indicated that OS was significantly longer in the low-risk group in the training, test, and entire TCGA cohorts (*p* < 0.05; Figures 2A–C). Notably, all the included lncRNAs, except *PRPRD-AS1*, were risk factors for poor prognosis in COAD patients. The heatmap shows that all of these lncRNAs, except *PRPRD-AS1*, were overexpressed in high-risk groups because only *PRPRD-AS1* was a protective factor (Figures 2D–F). The AUCs of the ROC in the three cohorts were acceptable for the lncRNA prognostic signature. Only the AUC at 1 year in the test cohort did not reach 0.7 (Figures 2G–I). Compared with the prognostic models of Li and Jin, our GIlncPS had the best performance (AUC = 0.735, 0.745, and 0.741, respectively; Figures 2J–L). Independent prognostic analysis indicated that old age, advanced stage, and occurrence of metastasis were significantly related to poor prognosis, and a low-risk score was significantly related to good prognosis (*p* < 0.05; Figures 3A,B). Figure 3C shows the nomogram for clinical use. The calibration curve demonstrated the reliability of the nomogram (Figure 3D). We also compared TMB/MSI and TME infiltration and functions between the high- and low-risk groups. However, the results differed from those of hierarchical clustering. As shown in Supplementary Figures 1A–H, all ICI outcome predictive biomarkers, except MSI, were not significantly different between the two groups, indicating that our prognostic signature behaved poorly in predicting genome instability, TME infiltration, and ICI response rates for COAD patients. Furthermore, Supplementary Figure 1I shows that the mutation rate of *BRAF* was higher in the high-risk group (19% vs. 11%). The prognostic ability of the GIlncPS was better than that of *BRAF* (*p* < 0.01; Supplementary Figure 1J). The OS in the high-risk groups was always lower than that in the low-risk groups, regardless of the *BRAF* mutation type. To conclude, we constructed a GIlncPS with strong prognostic ability, but it could not predict the TME infiltration and ICI response rates for COAD patients.

### Analysis of Mutation Profile and Identification of Hub-Long Noncoding RNAs in Clusters and Risk Groups

**FIGURE 4:**
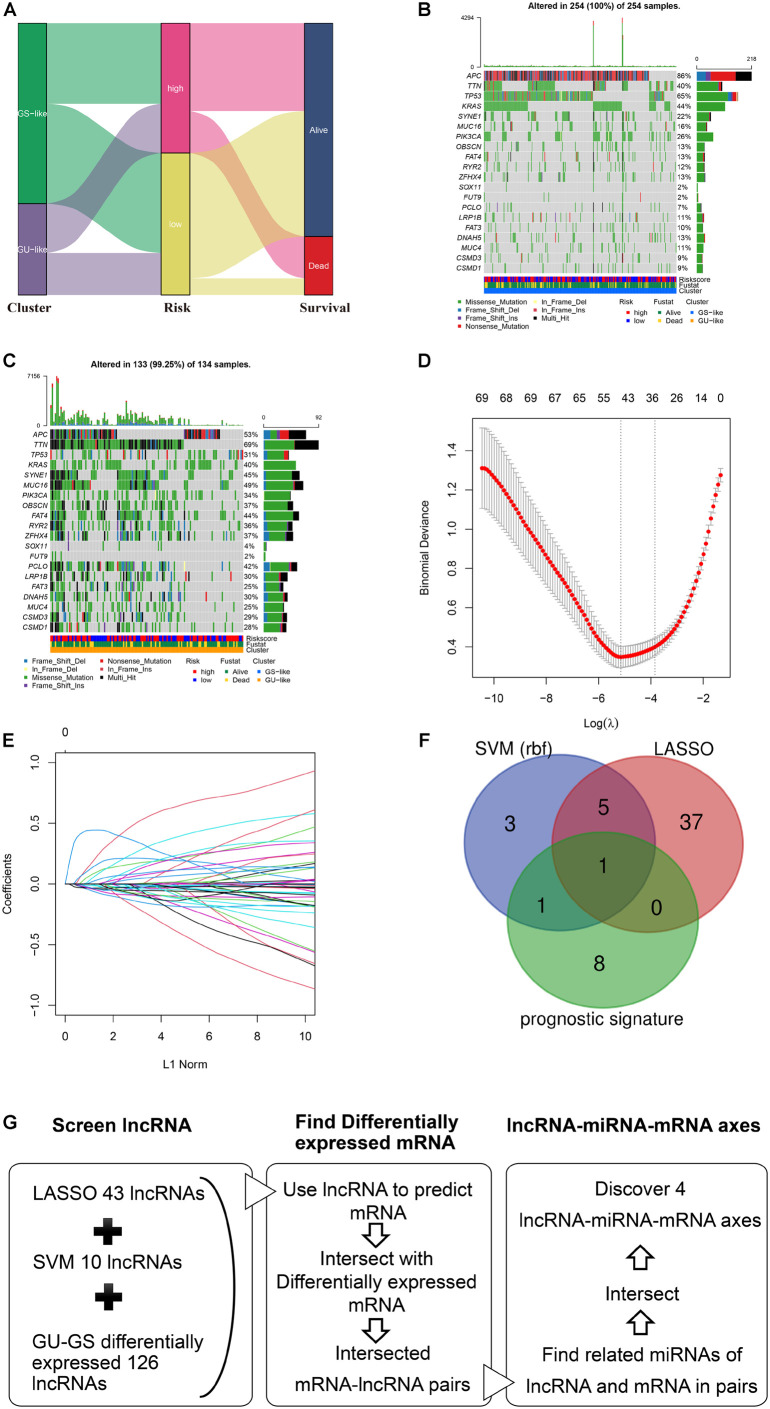
Analysis of mutation profile and identification of hub-lncRNAs in genome stable (GS)- and genome unstable (GU)-like clusters. **(A)** Sankey plot of the relationship among GS- and GU-like clusters, risk groups, and survival status. The mutation profile of GS-like cluster **(B)** and GU-like cluster **(C)**. **(D)** The selection of λ in least absolute shrinkage and selection operator regression. **(E)** The selection of L1 norm in LASSO regression. **(F)** The Venn plot of hub-lncRNA in SVM (support vector machine), LASSO, or prognostic signature. **(G)** Screening process of the lncRNA, miRNA, mRNA included in the ceRNA network.

The Sankey plot showed the relationship among the GS-and GU-like clusters, risk groups, and survival status (Figure 4A). The results showed no significant difference between the clusters and risk groups, and the death proportion in the high-risk group was higher. The top three genes with the highest mutation frequencies in the GS-like cluster were *APC* (86%), *TNN* (40%), and *TP53* (65%) (Figure 4B), whereas those in the GU-like cluster were *APC* (53%), *TTN* (69%), and *TP53* (31%) (Figure 4C). In general, the mutation frequency in the GU-like cluster was higher than that in the GS-like group, and the frequencies of multi-hit and frame-shift mutations were much higher. LASSO and SVM algorithms were utilized to extract hub-lncRNAs in GS- and GU-clusters for further investigation. We selected the minimum value of λ, a penalty parameter of 0.1, and the L1 norm of the default (Figures 4D,E). Here, 43 lncRNAs were screened using LASSO regression. The average weight of each lncRNA acquired from the 10-fold test SVM algorithm is provided in Supplementary Table 5. The cut-off value was set at | 0.28|. The Venn plot of the selection process of hub-lncRNA among SVM, LASSO, and the prognostic signature is presented in Figure 4F. In total, 55 hub lncRNAs were identified. The screening process of the lncRNAs, miRNAs, and mRNAs included in the ceRNA network is presented in Figure 4G.

### Genome Instability-Related Competing Endogenous RNA Network and Single-Gene Analysis

**FIGURE 5:**
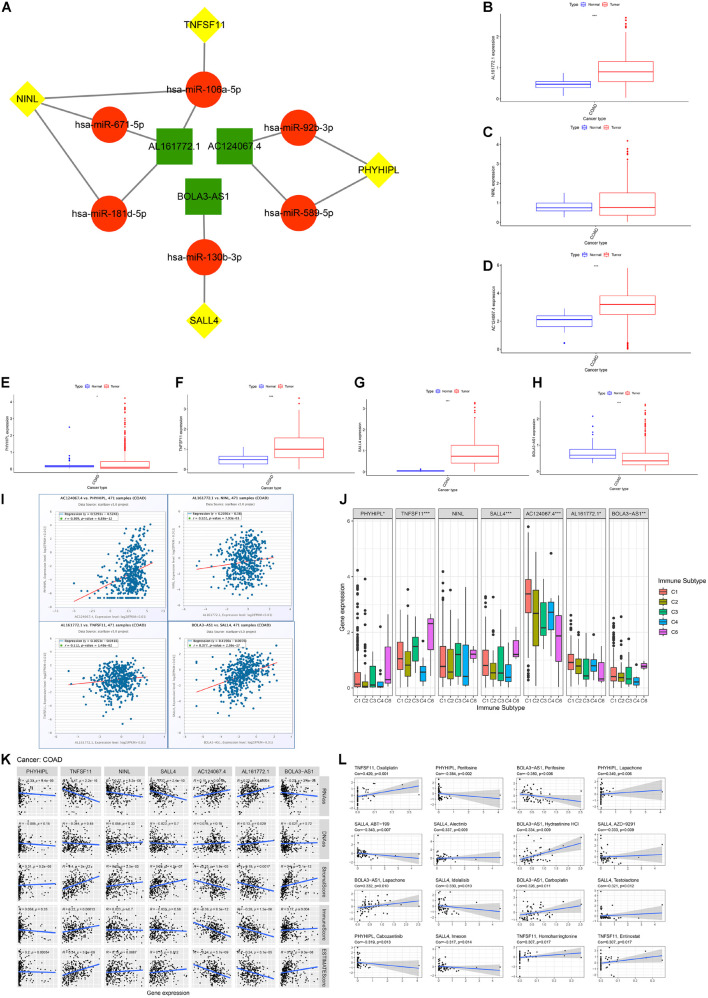
Competing endogenous RNA (ceRNA) network construction and immune, cancer stemness, and drug sensitivity analysis. **(A)** The genome instability-related ceRNA network in colon adenocarcinoma (COAD) based on ENCORI. **(B**–**H)** The differential expression analysis included three lncRNAs (*BOLA3-AS1*, *AC124067.4*, *AL161772.1*) and four target mRNAs (*NINL*, *SALL4*, *TNFSF11*, *PHYHIPL*) by Mann-Whitney *U* test. **(I)** The correlation between the expression of each lncRNA and its target mRNAs based on ENCORI. **(J)** The association between the seven gene expression and five immune subtypes in COAD. **(K)** The association between the seven-gene expression and cancer stemness based on mRNA expression (ssRNA) and DNA methylation patterns (DNAss); the association between the seven-gene expression and the ESTIMATE immune score, stromal score, and ESTIMATE score. **(L)** The association between the seven-gene expression between drug sensitivity based on the NCI60 database (top 16 ranked by correlation). *p < 0.05, ** *p* < 0.01, and ***p < 0.001.

The ENCORI database was utilized to predict the interactions between hub lncRNAs and their target mRNAs. The constructed ceRNA network is shown in Figure 5A and Table 2. Seven lncRNA-miRNA-mRNA pairs were found in the ceRNA network. To comprehensively understand these three lncRNAs (*BOLA3-AS1*, *AC124067.4*, and *AL161772.1*) and four target RNAs (*NINL*, *SALL4*, *TNFSF11*, *PHYHIPL*), we performed differential expression, cancer stemness, immune subtype, TME infiltration, and drug sensitivity analyses.

**TABLE 2:** The genome instability-related competing endogenous RNA network in COAD.

lncRNA	miRNA	mRNA
AL161772.1	hsa-miR-106a-5p	NINL
AL161772.1	hsa-miR-671a-5p	NINL
AL161772.1	hsa-miR-181d-5p	NINL
AL161772.1	hsa-miR-106a-5p	TNFSF11
AC124067.4	hsa-miR-92b-3p	PHYHIPL
AC124067.4	hsa-miR-589-5p	PHYHIPL
BOLA3-AS1	hsa-miR-130b-3p	SALL4

First, we compared the expression of seven genes between normal and tumor tissues using the Mann-Whitney *U* test in COAD. As shown in Figures 5B–H, *AC124067.4*, *AL161772.1*, *SALL4*, and *TNFSF11* were highly expressed in COAD, whereas *BOLA3-AS1* and *PHYHIPL* were expressed at low levels (*p* < 0.05). These results indicate that *AC124067.4*, *AL161772.1*, *SALL4*, and *TNFSF11* might promote COAD development and that *BOLA3-AS1* and *PHYHIPL* might inhibit its development (Figure 5I). As expected, the four lncRNA-mRNA expression patterns met the ceRNA criterion, especially *AC124067.4-PHYHIPL* (*r* = 0.309, *p* = 6.88e-12) and *BOLA3-AS1-SALL4* (*r* = 0.377, *p* = 2.56e-17). It has been reported that genome instability is closely related to tumor immunity. ANOVA was used to detect whether the expression of the seven genes was significantly related to the immune subtypes in COAD (Figure 5J). The results showed that six genes (*BOLA3-AS1*, *AC124067.4*, *AL161772.1*, *SALL4*, *PHYHIPL*, and *TNFSF11*) were significantly associated with the COAD immune subtype (*p* < 0.05), indicating that these seven genes could modulate tumor immune functions at the genetic level. These seven genes were also closely related to genome instability and immune-related genes (Supplementary Figure 2). All seven genes were positively related to *MLH1*, *PMS2*, and *NORAD* (*p* < 0.01). In addition, most genes (4/7, 57.1%) were negatively correlated with *PD-L1* (*CD274*; *p* < 0.05). These findings suggest that the high expression of these seven genes contributes to genomic integrity and predicts poor ICI outcomes. Moreover, these seven genes are closely related to COAD TME infiltration and function (Figure 5K). Most genes were positively related to stromal cell infiltration (5/7, 71.4%) and immune cell infiltration (4/7, 57.1%) and negatively related to tumor purity (5/7, 71.4%). According to the Spearman’s correlation between cancer stemness features and these seven genes, we found that most genes were negatively related to cancer stemness (5/7 (71.4%) for RNAss and 4/7 (57.1%) for DNAss; Figure 5K). Finally, drug sensitivity analysis was performed to explore the relationship between the seven genes and drug resistance. The results showed that the expression of these seven genes was positively associated with the sensitivity of drugs regulating DNA replication and synthesis. For example, *TNFSF11* expression was positively associated with sensitivity to oxaliplatin, a common platinum-based antineoplastic drug for colorectal cancer, which is believed to function by blocking the duplication of DNA (*r* = 0.429, *p* < 0.001). Meanwhile, some protein kinase inhibitors, such as perifosine and cobimetinib, were negatively related to these genes. In conclusion, we proposed a novel ceRNA network that modulates genome instability and found that these seven genes are involved in TME infiltration, cancer stemness, and drug resistance.

### Expression Pattern Validation by qPCR

**FIGURE 6:**
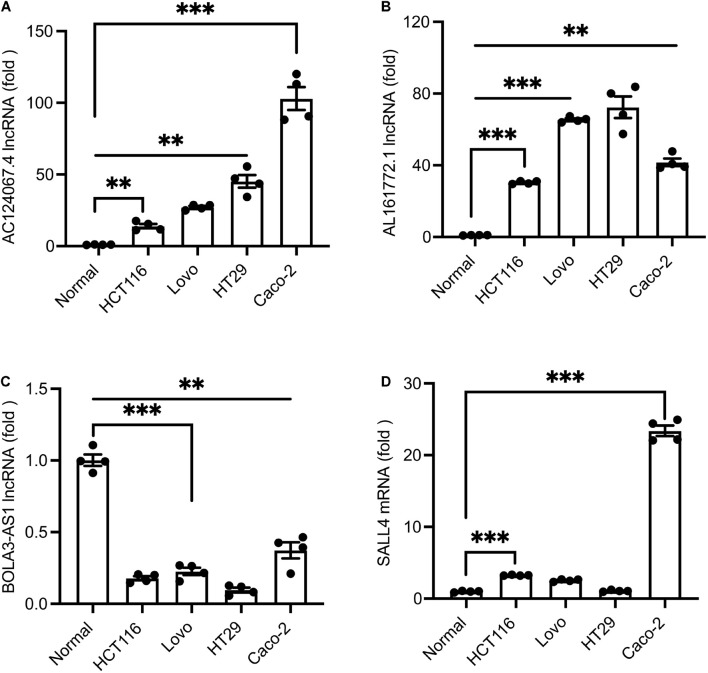
**(A–D)** qPCR verification of the expression levels of differential lncRNAs in COAD cell lines and normal cells. Values are shown as the mean ± SEM (*n* = 4). ***p < 0.001 and **p < 0.01 by Student’s *t*-test. Normal cells were employed as controls.

To ascertain these potential prognostic biomarkers, three lncRNAs (*AC124067.4*, *AL161772.1*, *BOLA3-AS1*) with significant expression alterations and one mRNA *SALL4* were further performed by qRT-PCR validation. The results showed that *AC124067.4*, *AL161772.1*, and *SALL4* gene levels were significantly increased in COAD cell lines (Figures 6A–D) whereas *BOLA3-AS1* (Figure 6) was significantly reduced, which was consistent with the above analysis. Taken together, the expressions of *AC124067.4*, *AL161772.1*, and *SALL4* were significantly upregulated, and their expression level was also associated with the OS rate in patients with COAD. These findings indicate the possibility of using the panel as a prognostic biomarker for COAD.

### Mutation Analysis

**FIGURE 7:**
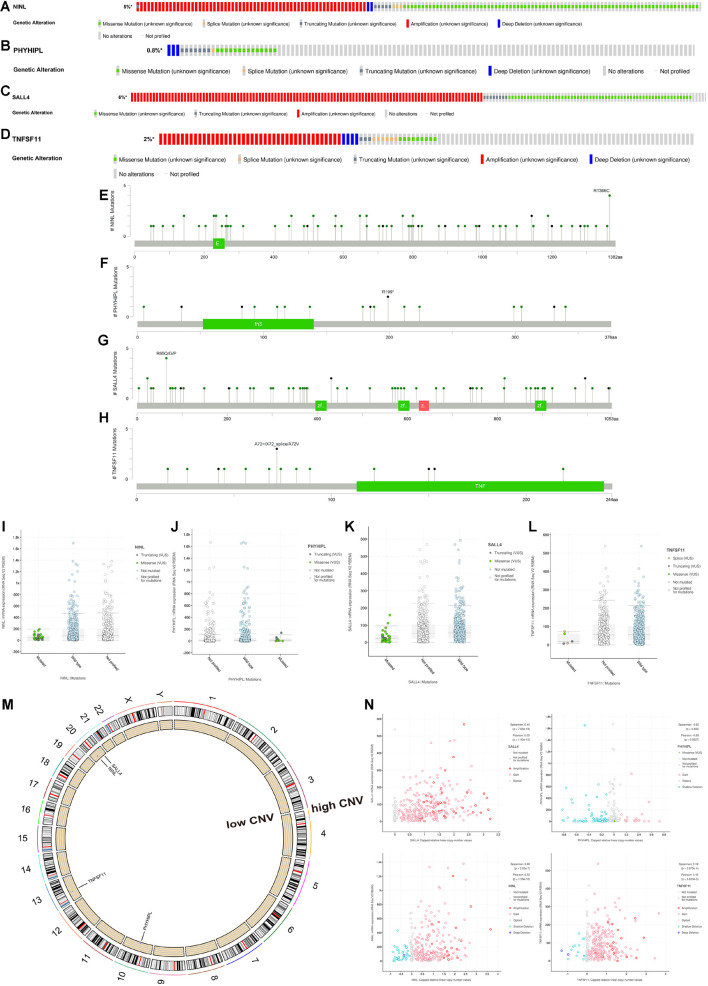
Somatic mutation and copy number variation (CNV) analysis of three lncRNAs (*BOLA3-AS1*, *AC124067.4*, *AL161772.1*) and four target mRNAs (*NINL*, *SALL4*, *TNFSF11*, *PHYHIPL*). The alteration rates and types of *NINL* **(A)**, *PHYHIPL* **(B)**, *SALL4* **(C)**, and *TNFSF11* **(D)**. The alteration sits of each gene on the coding protein domains for *NINL* **(E)**, *PHYHIPL* **(F)**, *SALL4* **(G)**, and *TNFSF11* **(H)**. The comparison of gene expression between wild type and mutated type for *NINL* **(I)**, *PHYHIPL* **(J)**, *SALL4* **(K)**, and *TNFSF11* **(L)**. **(M)** The location on a chromosome and the most common CNV status of the four target mRNAs. **(N)** The association between gene expression and CNV for *NINL*, *PHYHIPL*, *SALL4*, and *TNFSF11*.

First, we explored the rates of alteration and types with respect to the four target mRNAs. As shown in Figures 7A–D, the rates of *NINL*, *PHYHIPL*, *SALL4*, and *TNFSF11* alteration were 5%, 0.8%, 6%, and 2%, respectively. For *NINL* and *SALL4*, somatic mutations and CNVs accounted for almost half of these for each. Regarding *PHYHIPL* alterations, somatic mutations were predominant, whereas CNVs formed the majority of *TNFSF11* alterations. Missense mutations accounted for most somatic mutations among the four gene alterations, and amplification accounted for most CNVs among the *NINL*, *SALL4*, and *TNFSF11* alterations. The alteration sites of each target gene were also explored. Notably, all four gene alteration sites were dispersed across the entire gene, rather than clustering on specific domains, indicating that the four-gene alterations mainly affect mRNA expression but not the coded protein activity (Figures 7E–H). Therefore, we investigated whether alterations in the four genes were associated with their expression. As expected, three genes (*NINL*, *SALL4*, and *TNFSF11*) were expressed at low levels for mutated types, and the CNVs of the three genes were positively related to mRNA expression. However, the rate of *PHYHIPL* alterations was only 0.8%, and *PHYHIPL* expression was not related to its alteration type.

**FIGURE 8:**
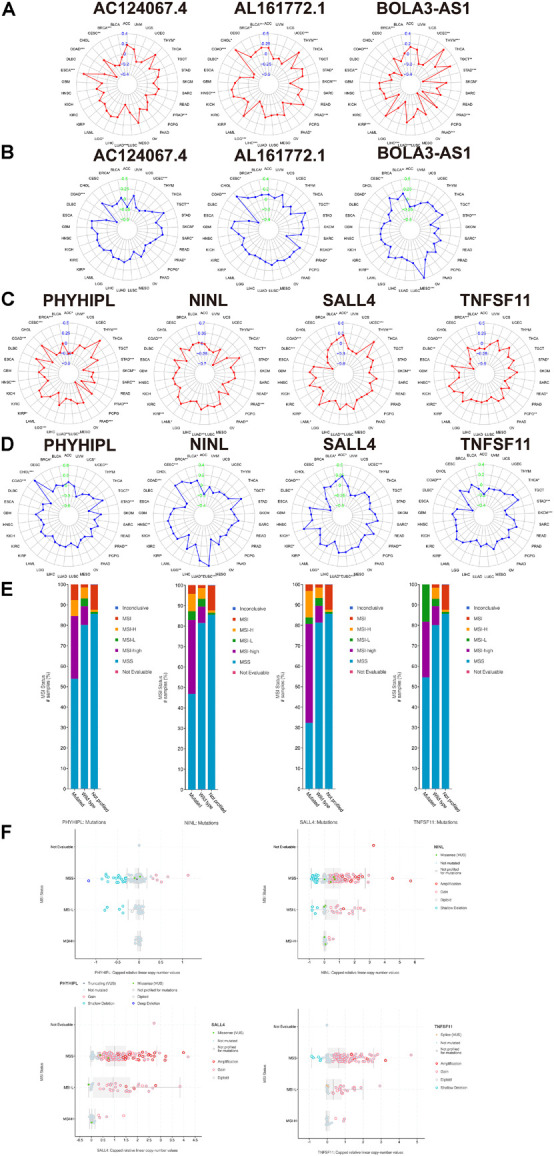
The association between the three lncRNAs (*BOLA3-AS1*, *AC124067.4*, and *AL161772.1*), four target mRNAs (*NINL*, *SALL4*, *TNFSF11*, and *PHYHIPL*), and the tumor mutational burden (TMB), microsatellite instability (MSI) score, and MSI status. **(A)** The association between the three lncRNA expression and the TMB in pan-cancer using Spearman’s correlation. **(B)** The association between the three lncRNA expression and the MSI score in pan-cancer. The association between the four target mRNAs and the TMB **(C)** and MSI score **(D)** in pan-cancer. **(E)** The comparison of the MSI status between the wild type and mutated type of the four target mRNAs (adjusted by each gene mutation rate) in COAD based on cBioPortal. **(F)** The association between the MSI status and the copy number variation of the four target mRNAs in COAD based on cBioPortal.

Microsatellite instability has become a recognized biomarker for immunotherapy and has been widely used in clinical practice. It is speculated that the seven genes proposed in this study could serve as clinically novel TMB and MSI biomarkers. As shown in Figures 8A–D, we explored the association between each gene and TMB/MSI in the pan-cancer database (the abbreviations are provided in Supplementary Table 6). Surprisingly, all the expression of all seven genes was negatively related to TMB and MSI scores in COAD (*p* < 0.05), indicating that these seven genes could maintain genomic integrity in COAD, which was particularly apparent for *AC124067.4* (correlation coefficient *R* reached approximately –0.4 for TMB and –0.5 for MSI) and *SALL4* (R reached approximately –0.3 for TMB and –0.35 for MSI; Figures 8A–D). Moreover, we compared MSI status between patients with mutated and wild-type disease using cBioPortal. The MSI-H proportions were greater in patients with the mutated type than in those with the wild type according to all four genes (Figure 8E). Especially, the MSI-H proportion reached almost 60% in patients with mutated *SALL4*, whereas only 10% of wild-type patients had MSI-H. We next explored the association between the seven CNVs and the MSI status. As shown in Figure 8F, the CNVs of *NINL*, *SALL4*, and *TNFSF11* were fewer in patients with MSI-H COAD. In conclusion, the seven genes proposed in this study were closely related to genome instability in COAD and could serve as novel immunotherapy outcome biomarkers.

## Discussion

This study presented primary work comprising a computational bioinformatics analysis to identify the critical genetic/epigenetic biomarkers from the genome instability-related ceRNA network involved in COAD. This study first provided a hierarchical clustering method to stratify patients for immunotherapy and a prognostic signature for clinical reference. Subsequently, genome insatiability-related ceRNA network genes were identified as critical regulators modulating TME infiltration, cancer stemness, and drug resistance. The identification of the lncRNA-miRNA-mRNA axes *AL161772.1-has-miR-671-5p* (*hsa-miR-181d-5p*, *has-miR-106a-5p*)-*NINL*, *AL161772.1-has-miR-106a-5p-TNFSF11*, *AC124067.4-hsa-miR-92b-3p* (*hsa-miR-589-5p*)-*PHYHIPL*, and *BOLA3-AS1-has-miR-130b-3p-SALL4* and the construction of a ceRNA network based on these four axes were the most significant findings of this study and are the main object of this discussion.

Among the four identified lncRNA-miRNA-mRNA signaling axes, *BOLA3-AS1-has-miR-130b-3p-SALL4* was considered the most valuable in modulating the tumorigenesis of COAD. *SALL4* and *BOLA3-AS1* were identified and defined as the most promising biomarkers, not only because of their respective high correlations with the same miRNA, which plays a role as a sponge and an intermediate, but also because of their highly consistent and synergic features. The explicit role of *SALL4* in COAD can be attributed to its pivotal role in cell proliferation, apoptosis, invasive migration, chemoresistance, and the maintenance of cancer stem cells (Zhang et al., 2015). First, *SALL4* is highly expressed and positively associated with *BOLA3-AS1* in COAD tumor tissues (Figure 5I), suggesting a positive regulation between them. Second, this work provides a novel viewpoint on how *SALL4* interacts with cancer cell stemness, thereby participating in tumor metastasis and progression. This work resulted in an estimate stemness score to contrast and evaluate the degree of stemness associated with genes (Figure 5K). *SALL4* and *BOLA3-AS1* both yielded impressive results in the multidimensional grading scale of the stemness score. *SALL4* has long been regarded as an essential modulator in maintaining embryonic stem cell (ESC) self-renewal and pluripotency (Miettinen et al., 2014). Considering that it is largely considered that ESCs and cancer stem cells share similar metabolic states, *SALL4* regulates the activation of several critical signaling pathways in stem cells by upregulating the expression of target genes in the Wnt/β-catenin pathway, and thus, its powerful regulatory role in cancer stemness might be explained in a similar way (Ma et al., 2006; Jang et al., 2015). Third, *SALL4* and *BOLA3-AS1* were associated with significant drug sensitivity (Figure 5l). Among the selected panels, filtered based on significance ranking, *SALL4*-related and *BOLA3-AS1*-related drugs accounted for the largest proportion. Through a literature search on PubMed, almost all related drugs are associated with their outstanding function as inhibitors of oxidative phosphorylation (de Witte et al., 2004; Burgeiro et al., 2013; Hammadi et al., 2015). Tan et al. (2019) reported the high-throughput screening of inhibitors of oxidative phosphorylation and *SALL4*, activating the transcription of genes that regulate oxidative phosphorylation to increase oxygen consumption, mitochondrial membrane potential, and ATP generation in cancer cells, which is the most predominant scientific finding on *SALL4*. Oxidative phosphorylation plays a critical role in the repair of DNA damage repair (dMMR). This is associated with the genome instability discussed in this article because dMMR is one of the culprits regulating genome instability. It is important to consider how *SALL4* was selected at first; specifically, it comprises one end of an axis, and at the other end is one of the most core genome instability-related lncRNAs, *BOLA3-AS1*. An entire ceRNA network was constructed on this basis. Therefore, although we still do not know the precise role of *SALL4* in COAD, the mechanism by which it influences genome instability and further promotes tumor progression *via* such a *BOLA3-AS1*-miRNA-*SALL4* axis is very likely to exist and could be explained.

Another important line of evidence when discussing these axes is the pan-cancer TMB/MSI correlation. For the *AL161772.1*-miRNA-*NINL* axes, both *AL161772.1* and *NINL* showed a significantly negative correlation with TMB and MSI in COAD, which indicates a function in maintaining genomic integrity. Similarly, *AL161772.1-has-miR-106a-5p-TNFSF11* and *AC124067.4-hsa-miR-92b-3p*(*hsa-miR-589-5p*)-*PHYHIPL* both reduce the TMB and MSI in COAD, decreasing the risk of alterations and genome instability.

Diverse degrees of mutations were noted for *NINL*, *TNFSF11*, and *PHYHIPL*. *NINL* and *TNFSF11* have relatively high genetic alteration rates, and the type is primarily amplification. The sites of *NINL* alterations were the coding protein domains, which were scattered and concentrated in segments. The main mutation site of *NINL* is on chromosome 22, which is associated with relatively lower CNV, which is the same as that for *SALL4*, but *SALL4* has a higher CNV, and the mutation rate of *PHYHIPL* is not high. The genetic alteration site was also not dense. It is reasonable to speculate that major alterations do not include *PHYHIPL* mutations.

Two major weaknesses of this study should be further improved. One is that even though a novel ceRNA mechanism, regulating certain lncRNA-miRNA-mRNA axes and cancer promotion in COAD, was suggested, whether these genes could play a synergistic role in regulating tumor progression needs to be confirmed. More work also needs to be done to provide more details on how these genes interact with each other at the cellular level, with validation based on a clinical cohort. Another limitation is that the crucial genes screened have been rarely reported and researched previously. Although it is easy to find clues relevant to this topic, it is difficult to find direct support of the importance of these genes in the literature. In fact, this finding underlies the importance of this work, which suggests valuable biomarkers for COAD treatment for further research.

This study constructed a prognostic signature of genome instability-related lncRNA and immunotherapy for clinical reference. It also provided a potential ceRNA mechanism through which lncRNAs play a role via specific lncRNA-miRNA-mRNA axes to participate in the process of cancer development.

## Data Availability Statement

The original contributions presented in the study are included in the article/Supplementary Material, further inquiries can be directed to the corresponding author/s.

## Author Contributions

ZR and ZW performed the data analysis and wrote the first draft. DG and HM revised the first and the second drafts. MC and YZ performed the experiments. YZ revised the second draft. JZ designed and supervised the study. All authors contributed to the article and approved the submitted version.

## Conflict of Interest

The authors declare that the research was conducted in the absence of any commercial or financial relationships that could be construed as a potential conflict of interest.

## Publisher’s Note

All claims expressed in this article are solely those of the authors and do not necessarily represent those of their affiliated organizations, or those of the publisher, the editors and the reviewers. Any product that may be evaluated in this article, or claim that may be made by its manufacturer, is not guaranteed or endorsed by the publisher.

## Footnotes

1
http://starbase.sysu.edu.cn

## Supplementary Material

Supplementary Figure 1 Genome instability and immune analysis of high- and low-risk groups. **(A)** Association between somatic mutation count and risk score. Comparison of tumor mutational burden **(B)**, microsatellite instability **(C)**, expression and mutation rate of four DNA mismatch repair protein genes (*MLH1*, *MSH2*, *MSH6*, and *PMS2*) **(D)**, and expression of six immune checkpoint-related genes (*CD274* (*PD-L1*), *CD80*, *PDCD1* (*PD-1*), *PDCD1LG2* (*PD-L2*), *CD86*, *CTLA4*) **(E)** between the two groups using the Mann-Whitney *U* test. Comparison of immune cell infiltration **(F)** and immune functions **(G)** of the two groups using single-sample gene set enrichment analysis (ssGSEA). ^∗^*p* < 0.05; ^∗∗^*p* < 0.01; and ^∗∗∗^*p* < 0.001. **(H)** Comparison of the mutation rate of *POLE* between the two groups using the Mann-Whitney *U*-test. **(I)** Comparison of BRAF mutation rates. **(J)** Survival analysis among *BRAF* mutated/high-risk, *BRAF* mutated/low-risk, *BRAF* wild/high-risk, *BRAF* wild/low-risk groups by Kaplan-Meier curve and log-rank test.

Supplementary Figure 2 Supplemental gene correlation analysis of the three lncRNAs (*BOLA3-AS1*, *AC124067.4*, and *AL161772.1*) and four target mRNAs (*NINL*, *SALL4*, *TNFSF11*, and *PHYHIPL*). **(A)** The association between the expression of seven genes and genome instability and immune-related genes (*PDCD1*, *CD274*, *PDCD1LG2*, *CTLA4*, *CD80*, *CD86*, *MSH2*, *MLH1*, *PMS2*, *MSH6*, *NOARD*, *GUARDIN*) by Spearman’s correlation. **(B)** Correlation between the expression of each miRNA and its paired lncRNAs based on ENCORI. **(C)** Correlation between the expression of each miRNA and its paired mRNAs based on ENCORI. ^∗^*p* < 0.05; ^∗∗^*p* < 0.01; and ^∗∗∗^*p* < 0.001.

Supplementary Figure 3 The association between the three lncRNAs (*BOLA3-AS1*, *AC124067.4*, and *AL161772.1*), four target mRNAs (*NINL*, *SALL4*, *TNFSF11*, *PHYHIPL*), and each specific type of immune cell based on the CIBERSORT database.
