# Supplementary figures and images for: Genome Instability and Long Noncoding RNA Reveal Biomarkers for Immunotherapy and Prognosis and Novel Competing Endogenous RNA Mechanism in Colon Adenocarcinoma
Source: Front Cell Dev Biol. 2021 Oct 20;9:740455. doi: 10.3389/fcell.2021.740455 (PMC8564000; doi:10.3389/fcell.2021.740455)

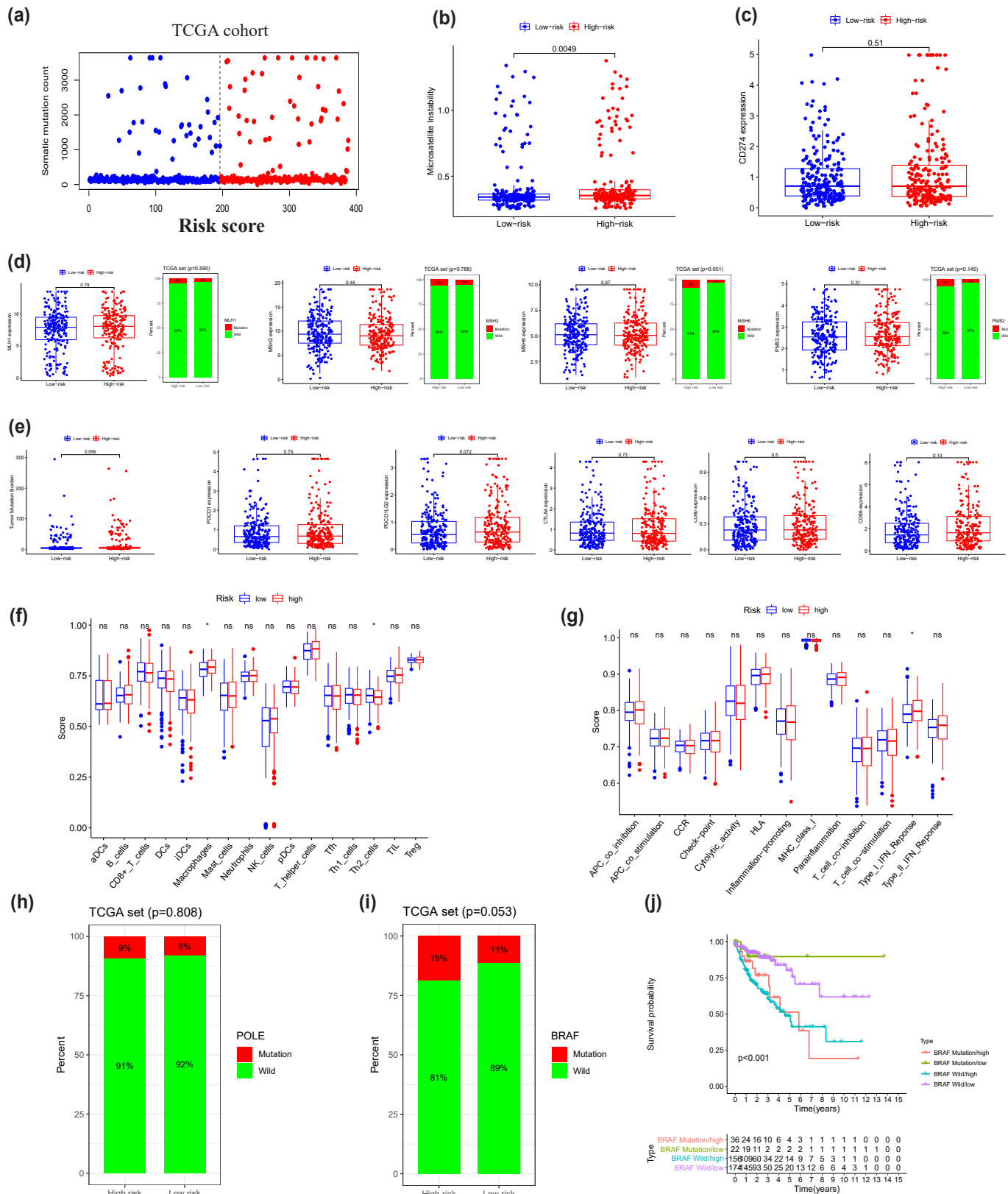

Supplement: Supplementary Figure 1 — Genome instability and immune analysis of high- and low-risk groups. (A) Association between somatic mutation count and risk score. Comparison of tumor mutational burden (B), microsatellite instability (C), expression and mutation rate of four DNA mismatch repair protein genes (MLH1, MSH2, MSH6, and PMS2) (D), and expression of six immune checkpoint-related genes (CD274 (PD-L1), CD80, PDCD1 (PD-1), PDCD1LG2 (PD-L2), CD86, CTLA4) (E) between the two groups using the Mann-Whitney U test. Comparison of immune cell infiltration (F) and immune functions (G) of the two groups using single-sample gene set enrichment analysis (ssGSEA). ∗p < 0.05; ∗∗p < 0.01; and ∗∗∗p < 0.001. (H) Comparison of the mutation rate of POLE between the two groups using the Mann-Whitney U-test. (I) Comparison of BRAF mutation rates. (J) Survival analysis among BRAF mutated/high-risk, BRAF mutated/low-risk, BRAF wild/high-risk, BRAF wild/low-risk groups by Kaplan-Meier curve and log-rank test. [file Data_Sheet_1.PDF]

(a)

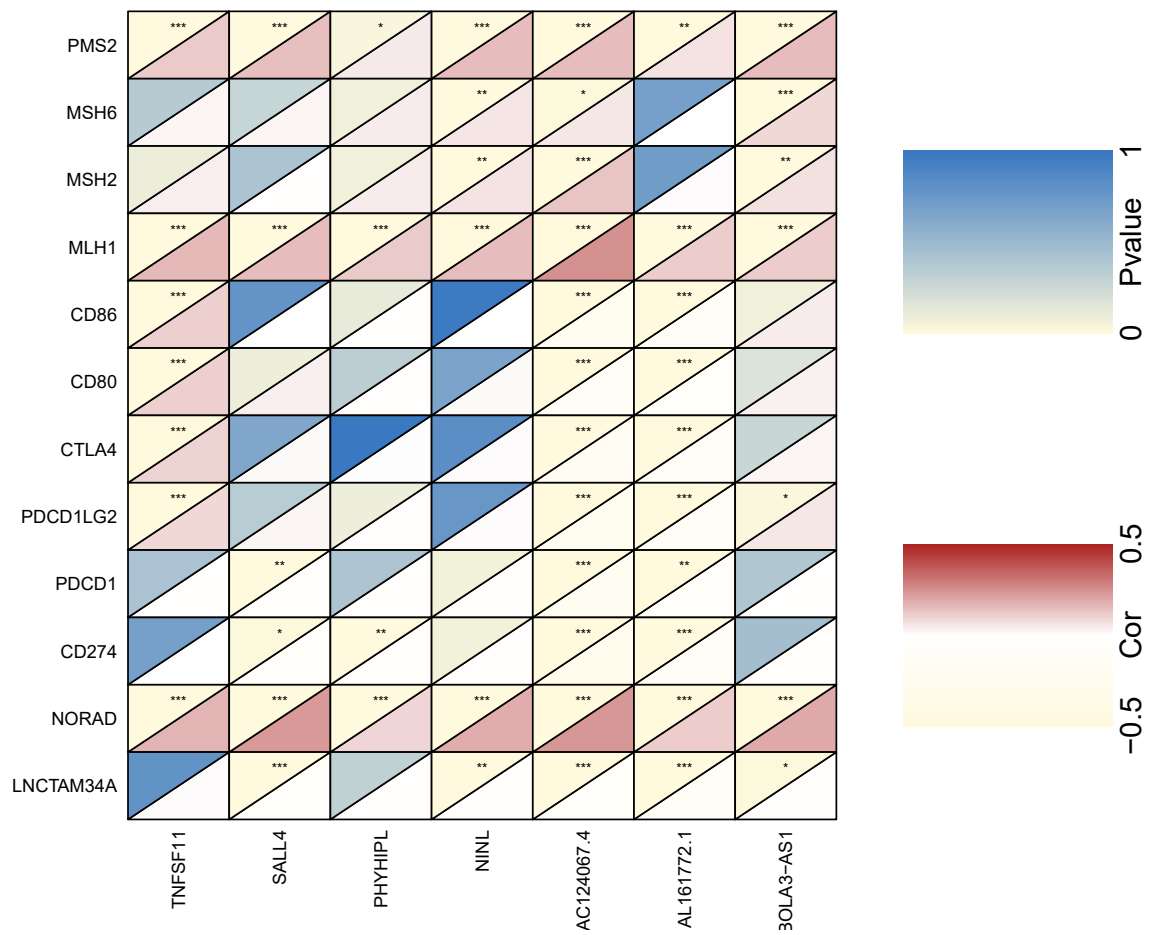

(b) IncRNA-miRNA

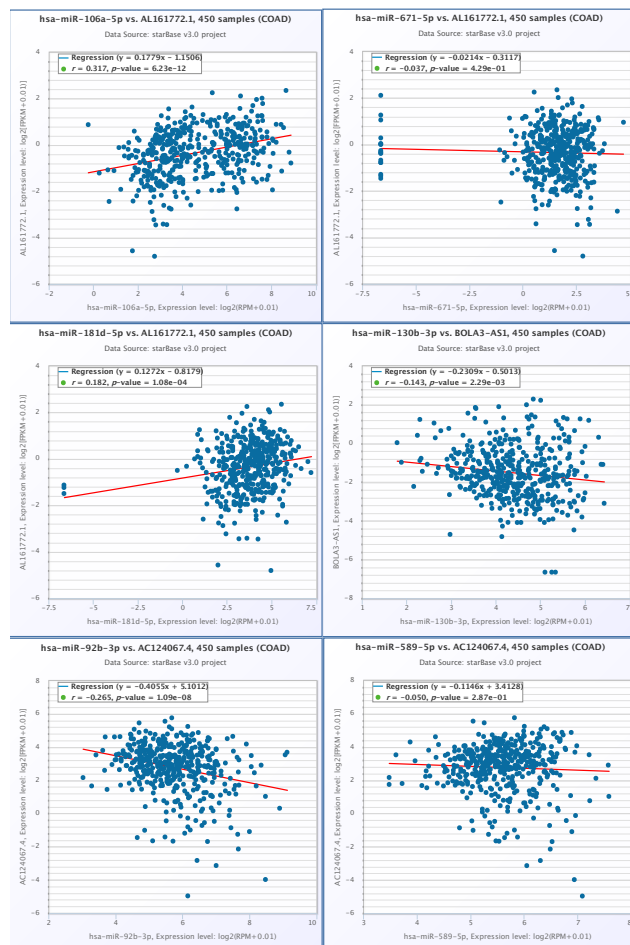

(c) miRNA-mRNA

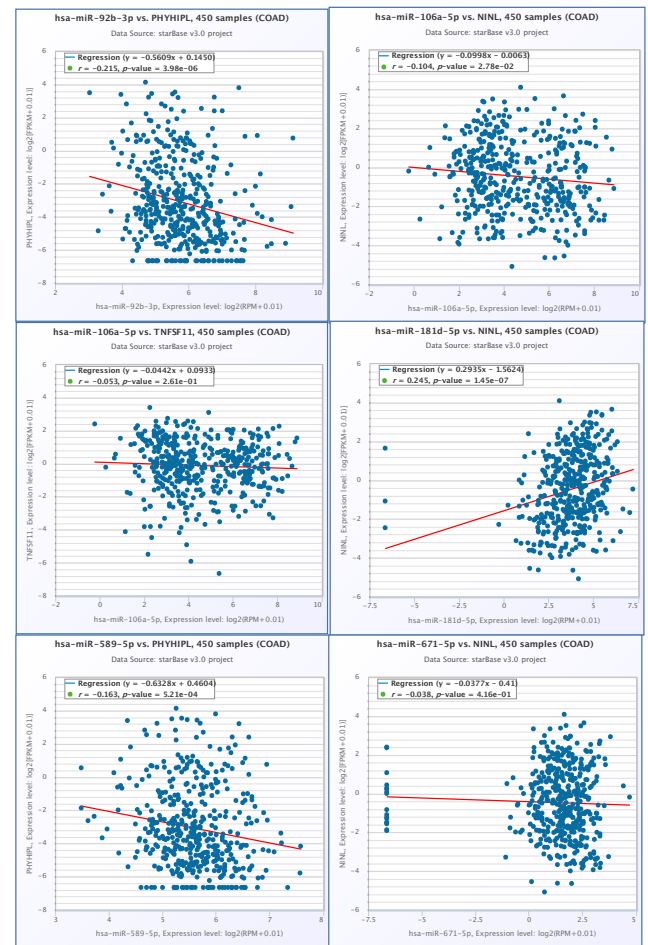

Supplement: Supplementary Figure 2 — Supplemental gene correlation analysis of the three lncRNAs (BOLA3-AS1, AC124067.4, and AL161772.1) and four target mRNAs (NINL, SALL4, TNFSF11, and PHYHIPL). (A) The association between the expression of seven genes and genome instability and immune-related genes (PDCD1, CD274, PDCD1LG2, CTLA4, CD80, CD86, MSH2, MLH1, PMS2, MSH6, NOARD, GUARDIN) by Spearman’s correlation. (B) Correlation between the expression of each miRNA and its paired lncRNAs based on ENCORI. (C) Correlation between the expression of each miRNA and its paired mRNAs based on ENCORI. ∗p < 0.05; ∗∗p < 0.01; and ∗∗∗p < 0.001. [file Data_Sheet_2.PDF]

(a) Cancer: COAD

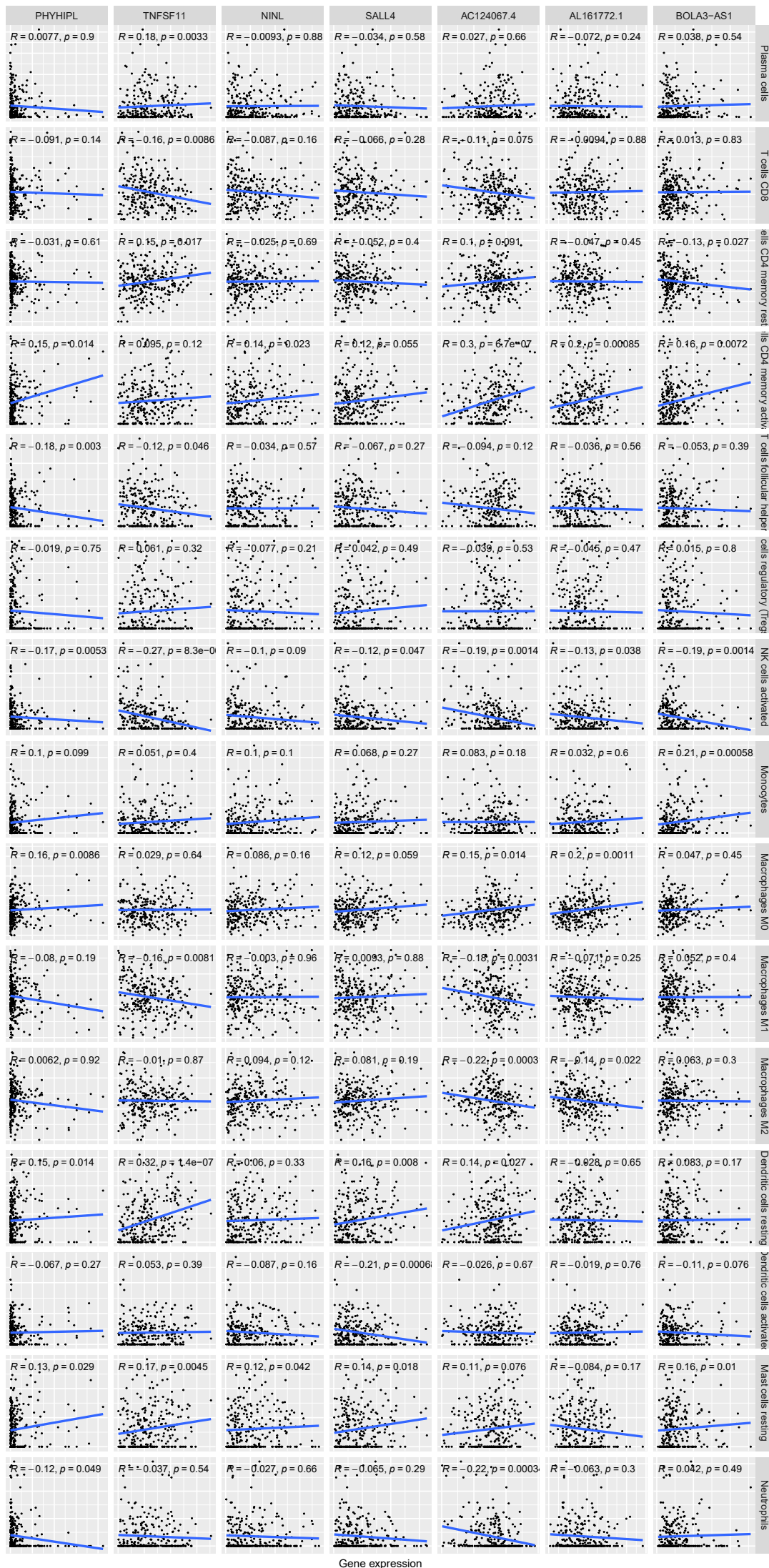

Gene expression

Supplement: Supplementary Figure 3 — The association between the three lncRNAs (BOLA3-AS1, AC124067.4, and AL161772.1), four target mRNAs (NINL, SALL4, TNFSF11, PHYHIPL), and each specific type of immune cell based on the CIBERSORT database. [file Data_Sheet_3.PDF]
